# Construction of artificial neural network (ANN) based on predictive value of prognostic nutritional index (PNI) and neutrophil-to-lymphocyte ratio (NLR) in patients with cervical squamous cell carcinoma

**DOI:** 10.1097/MD.0000000000037680

**Published:** 2024-04-05

**Authors:** Xiaohao Li, Chaoyang Ou, Aiqin He

**Affiliations:** a Department of Gynecology and Obstetrics, The People’s Hospital of Tongzhou District, Nantong, Jiangsu, China; b Department of Gynecological Oncology, Nantong Tumor Hospital, The Affiliated Tumor Hospital of Nantong University, Nantong, Jiangsu, China.

**Keywords:** cervical squamous cell carcinoma, neutrophil-to-lymphocyte ratio (NLR), overall survival (OS), prognosis, prognostic nutritional index (PNI)

## Abstract

To explore the analytical worth of prognostic nutritional index (PNI) and neutrophil-to-lymphocyte ratio (NLR) in patients with cervical squamous cell carcinoma. The clinical data of 539 patients with cervical cancer in the Affiliated Tumor Hospital of Nantong University from December 2007 to October 2016 were analyzed retrospectively. The ROC is used to select the best cutoff values of PNI and NLR, which are 48.95 and 2.4046. Cox regression analysis was used for univariate and multivariate analysis. Survival differences were assessed by Kaplan–Meier (KM) survival method. Finally, a 3-layer artificial neural network (ANN) model is established. In cervical squamous cell carcinoma, the KM survival curve showed that the overall survival (OS) rate of high-level PNI group was significantly higher than that of low-level PNI group (*P* < .001), while the OS rate of low-level NLR group was significantly higher than that of high-level NLR group (*P* = .002). In non-squamous cell carcinoma, there was no significant difference in OS between the 2 groups (*P* > .005). According to Cox multivariate analysis, preliminary diagnosed PNI and NLR were independent prognostic factors of cervical squamous cell carcinoma (*P* < .001, *P* = .008), and pathological type and International Federation of Gynecology and Obstetrics (FIGO) stage also had a certain impact on tumor progression (*P* = .042, *P* = .048). The increase of PNI and the decrease of NLR will help patients with cervical squamous cell carcinoma live longer. ANN showed that PNI and NLR were of great importance in predicting survival. Preoperative PNI and NLR are independent predictors of cervical squamous cell carcinoma patients related to clinicopathological features, and have particular value in judging prognosis.

## 1. Introduction

The incidence rate of cervical cancer ranks fourth among female malignant tumors, and is the fourth leading cause of cancer mortality in women. In 2020, there were 600,000 cervical cancer cases worldwide, with a year-on-year growth rate of 5.3%, and 340,000 deaths, with a year-on-year growth rate of 9.3%. From this, the incidence rate and mortality rate of cervical cancer show a trend of increasing year by year, especially in low- and middle-income developing countries.^[1,2]^ Cervical cancer includes 3 common pathological subtypes: squamous cell carcinoma, adenocarcinoma and adenosquamous carcinoma. Among them, squamous cell carcinoma is the most common pathological type, accounting for about 75% of all subtypes.^[3]^ The pathogenesis of squamous cell carcinoma is a continuous development process from cervical intraepithelial neoplasia to invasive cancer.^[4]^ Many patients miss the best treatment period due to the slow onset of cervical cancer and no obvious symptoms in the early stage. Therefore, it is of great significance to find markers to predict the prognosis of cervical squamous cell carcinoma, which can help clinicians provide personalized treatment.

Increasing evidence shows that the prognosis of cancer is related not only to exogenous factors, but also to endogenous factors, such as nutritional status^[5,6]^ and systemic inflammation.^[7,8]^ Relevant nutritional indicators, such as prognostic nutritional index (PNI), is value based on serum albumin concentration and peripheral blood lymphocyte count. PNI index was originally proposed by Buzby et al to predict nutritional index and surgical risk.^[9]^ In recent years, more and more studies have shown that low-level of PNI is a poor prognostic factor for many cancers (non-small cell lung cancer, nasopharyngeal carcinoma, gastric cancer, pancreatic cancer, etc).^[10–13]^ Inflammation is 1 of the 6 biological abilities of tumor development and a sign of cancer. It is related to the occurrence, development,^[14]^ metastasis and prognosis of cancer.^[15]^ Inflammatory status can be reflected by corresponding serum indexes, such as C-reactive protein,^[16]^ erythrocyte distribution width,^[17]^ erythrocyte sedimentation rate,^[18]^ etc. The neutrophil-to-lymphocyte ratio (NLR) is considered to be an indicator of inflammatory response in cancer patients.^[19]^ Because of its convenient availability, it has high clinical significance.^[20]^

However, so far, there is no systematic study on the predictive value of PNI and NLR in cervical squamous cell carcinoma. Therefore, this retrospective study evaluated PNI and NLR as new prognostic markers of cervical squamous cell carcinoma, which provides new insights for the understanding of cervical squamous cell carcinoma.

## 2. Materials and methods

### 2.1. Patients

We systematically reviewed and analyzed the clinical data of patients with cervical cancer diagnosed in the Affiliated Tumor Hospital of Nantong University from December 2007 to October 2016. The diagnostic criteria were determined according to the latest NCCN guidelines for the diagnosis and treatment of cervical cancer. The inclusion criteria were as follows: Complete medical record data and follow-up information; According to pathology, it is only diagnosed as cervical cancer; Routine plasma test within 1 week before operation, including serum albumin, neutrophil and lymphocyte count. According to some force majeure factors, the exclusion criteria are as follows: Incomplete medical records and follow-up data; Have a history of cancer or currently have multiple cancers; Suffering from diseases that may affect serum albumin, neutrophil and lymphocyte counts. This study was approved by the ethics committee of the Affiliated Tumor Hospital of Nantong University and carried out in accordance with relevant guidelines and regulations. Since this study is a retrospective analysis based on the existing clinical data of patients and does not involve clinical trials or the use of human tissue samples, informed consent is not required for this study. The study was conducted in conjunction with the Helsinki Declaration (revised in 2013). Finally, 539 eligible patients were registered in this medical center.

### 2.2. Data collection and calculation

We collected patient data, including age, pathological type, tissue grade, depth of invasion, tumor size, preoperative clinical serological indicators (serum albumin, neutrophil and lymphocyte counts). The calculation formula of preoperative PNI and NLR is: PNI = serum albumin (g/L) + 5 * lymphocyte count (10^9/L); NLR = neutrophil (10^9/L)/lymphocyte count (10^9/L). Overall survival (OS) was defined as the time from surgery to death or, if the patient was still alive, to the last follow-up. The final follow-up date was April 10, 2023.

### 2.3. Statistical analysis

All statistical analyses were implemented by IBM SPSS software (version 25.0). The subject operating curve ROC was used to evaluate the predictive performance of PNI and NLR, and the maximum Youden index (sensitivity + specificity-1) was selected as the best critical value. Chi square test was used to compare categorical variables, and KM survival method was used to evaluate survival differences. Univariate and multivariate Cox proportional hazards regression models were used to determine independent prognostic factors. The results were shown as hazard ratio (HR) and 95% confidence interval (CI). Finally, a 3-layer ANN (input layer, hidden layer and output layer) is established. 70% of the queue is randomly selected for modeling, and the remaining 30% is used for verification. Values of *P* < .05 were considered statistically important.

## 3. Results

### 3.1. Patient characteristics

A total of 539 patients with cervical cancer were included in our study. Table 1 summarizes the detailed clinical factors of patients. There were 245 patients ≤ 55 years old and 294 patients > 55 years old. According to the pathological type, it can be divided into cervical squamous cell carcinoma, adenocarcinoma and adenosquamous cell carcinoma, accounting for 90.72%, 6.31% and 2.97% respectively. According to International Federation of Gynecology and Obstetrics (FIGO) stage, 355 patients (65.86%) were stage I to IIA tumors and 184 patients (34.14%) were stage IIB to IV tumors. 322 cases (59.74%) were classified as grade I and grade II, while 217 cases (40.26%) were classified as grade III. The degree of invasion was divided into ≤1/2 and >1/2, accounting for 47.12% and 52.88%, respectively. There were 262 patients with tumor size ≤3 cm, and 277 patients with tumor size >3 cm. The median values of PNI and NLR were 53.10 and 2.49, respectively.

**Table 1:** Characteristics of included patients (n = 539).

Characteristics	Value (range or n%)
Number of patients	539 (100.00)
Age	
≤55 yr	245 (45.45)
>55 yr	294 (54.55)
Pathological type	
Squamous carcinoma	489 (90.72)
Adenocarcinoma	34 (6.31)
Adenosquamous carcinoma	16 (2.97)
FIGO stage	
I–IIA	355 (65.86)
IIB–IV	184 (34.14)
Histologic grade	
I–II	322 (59.74)
III	217 (40.26)
Depth of invasion	
≤1/2	254 (47.12)
>1/2	285 (52.88)
Tumor size	
≤3 cm	262 (48.61)
>3 cm	277 (51.39)
PNI	53.10 (31.80–68.10)
NLR	2.49 (0.00–22.70)

FIGO = International Federation of Gynecology and Obstetrics, NLR = neutrophil-to-lymphocyte ratio, PNI = prognostic nutritional indicators.

### 3.2. Relationship between PNI and NLR with patient characteristics

**Figure 1.**
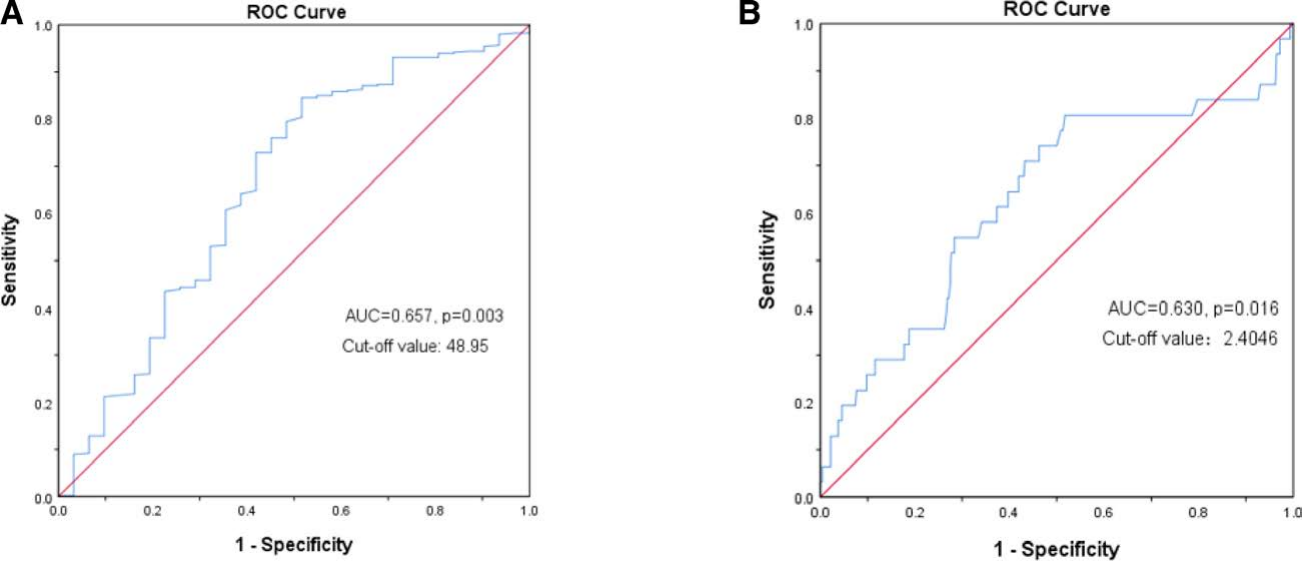
Analysis of ROC curves. ROC based on (A) PNI and (B) NLR for overall survival. NLR = neutrophil-to-lymphocyte ratio, PNI = prognostic nutritional index.

We used ROC curve analysis to determine the predictive significance of PNI and NLR pretreatment values. With OS as the endpoint, the area under the ROC curve of PNI was 0.657 (*P* = .003). The area under the ROC curve of NLR was 0.630 (*P* = .016). According to the maximum value of Yoden index (sensitivity + specificity-1), the best cutoff values of PNI and NLR are 48.95 and 2.4046, respectively. 109 patients (20.22%) with PNI < 48.95 and 430 patients (79.78%) with PNI ≥ 48.95 were divided into low PNI group and high PNI group. 254 (47.12%) patients with NLR ≤ 2.4046 and 285 (52.88%) patients with NLR > 2.4046 were divided into low NLR group and high NLR group (Fig. 1A–B).

Table 2 shows the relationship between preoperative PNI and NLR and clinicopathological parameters in patients with cervical cancer. According to the analysis, there were significant differences in pathological type (*P* = .000), FIGO stage (*P* = .016) and tumor size (*P* = .038) between high and low preoperative PNI groups. However, there were significant differences in pathological type (*P* = .000) and FIGO stage (*P* = .039) between high and low NLR groups.

**Table 2:** Relationship between preoperative PNI and NLR and the clinical-pathological data of patients.

Characteristic	Total number N (%)	PNI, N (%)			NLR, N(%)		
		<48.95	≥48.95	*P* values	≤2.4046	>2.4046	*P* values
Age				.584			.801
≤55 yr	245 (45.45)	47 (8.72)	198 (36.73)		114 (21.15)	131 (24.30)	
>55 yr	294 (54.55)	62 (11.51)	232 (43.04)		140 (25.98)	154 (28.57)	
Pathological type				.000***			.000***
Squamous carcinoma	489 (90.72)	86 (15.95)	403 (74.77)		231 (42.86)	258 (47.86)	
No-squamous carcinoma	50 (9.28)	23 (4.27)	27 (5.01)		23 (4.27)	27 (5.01)	
FIGO stage				.016*			.039*
I–IIA	355 (65.86)	78 (14.47)	277 (51.39)		172 (31.91)	183 (33.95)	
IIB–IV	184 (34.14)	31 (5.75)	153 (28.39)		82 (15.21)	102 (18.93)	
Histologic grade				.292			.545
I–II	322 (59.74)	71 (13.17)	251 (46.57)		150 (27.83)	172 (31.91)	
III	217 (40.26)	37 (6.86)	180 (33.40)		93 (17.25)	124 (23.01)	
Depth of invasion				.644			.847
≤1/2	254 (47.12)	42 (7.79)	212 (39.33)		123 (22.82)	131 (24.30)	
>1/2	285 (52.88)	56 (10.39)	229 (42.49)		134 (24.86)	151 (28.02)	
Tumor size				.038*			.564
≤3 cm	262 (48.61)	42 (7.79)	220 (40.82)		121 (22.45)	141 (26.16)	
>3 cm	277 (51.39)	58 (10.76)	219 (40.63)		119 (22.08)	158 (29.31)	

FIGO = International Federation of Gynecology and Obstetrics, N = number, NLR = neutrophil-to-lymphocyte ratio, PNI = prognostic nutritional indicators.

### 3.3. Univariate and multivariate analysis results

Univariate analysis showed age (HR 1.620, 95% confidence interval [CI]: 0.806–3.257, *P* = .017), pathological type (HR 0.319, 95% confidence interval 0.145–0.703, *P* = .005), FIGO stage (HR 1.294, 95% CI 0.658–2.546, *P* = .035), tissue grade (HR 0.633, 95% CI: 0.223–1.796, *P* = .039), PNI (HR 4.554, 95% CI: 2.346–8.839, *P* < .001) and NLR (HR 0.267, 95% CI: 0.116–0.610, *P* = .002) were independent prognosis factors of cervical cancer (Table 3).

**Table 3:** Univariate and multivariate analyses of survival in cervical cancer patients.

Characteristic	Univariate		Multivariate	
	HR (95%CI)	*P* values	HR (95%CI)	*P* values
Age				
≤55 yr	Ref			
>55 yr	1.620 (0.806–3.257)	.017*		
Pathological type				
Squamous carcinoma	Ref		Ref	
No-squamous carcinoma	0.319 (0.145–0.703)	.005**	0.431 (0.191–0.971)	.042*
FIGO stage				
I–IIA	Ref		Ref	
IIB–IV	1.294 (0.658–2.546)	.035*	1.435 (0.726–2.840)	.048*
Histologic grade				
I–II	Ref			
III	0.633 (0.223–1.796)	.039*		
Depth of invasion				
≤1/2	Ref			
>1/2	0.881 (0.237–3.281)	.850		
Tumor size				
<3 cm	Ref			
≥3 cm	1.270 (0.539–2.990)	.585		
PNI				
<48.95	Ref		Ref	
≥48.95	4.554 (2.346–8.839)	<.001***	3.312 (1.661–6.602)	<.001***
NLR				
≤2.4046	Ref		Ref	
>2.4046	0.267 (0.116–0.610)	.002**	0.318 (0.138–0.737)	.008**

FIGO = International Federation of Gynecology and Obstetrics, HR = hazard ratio, NLR = neutrophil-to-lymphocyte ratio, PNI = prognostic nutritional index.

In multivariate analysis, pathological type (HR 0.431, 95% CI: 0.191–0.971, *P* = .042), FIGO stage (HR 1.435, 95% CI: 0.726–2.840, *P* = .048), PNI (HR 3.313, 95% CI: 1.661–6.602, *P* < .001) and NLR (HR 0.318, 95% CI: 0.138–0.737, *P* = .008) were significant predictors of the prognosis of cervical cancer (Table 3).

### 3.4. Survival analysis

**Figure 2.**
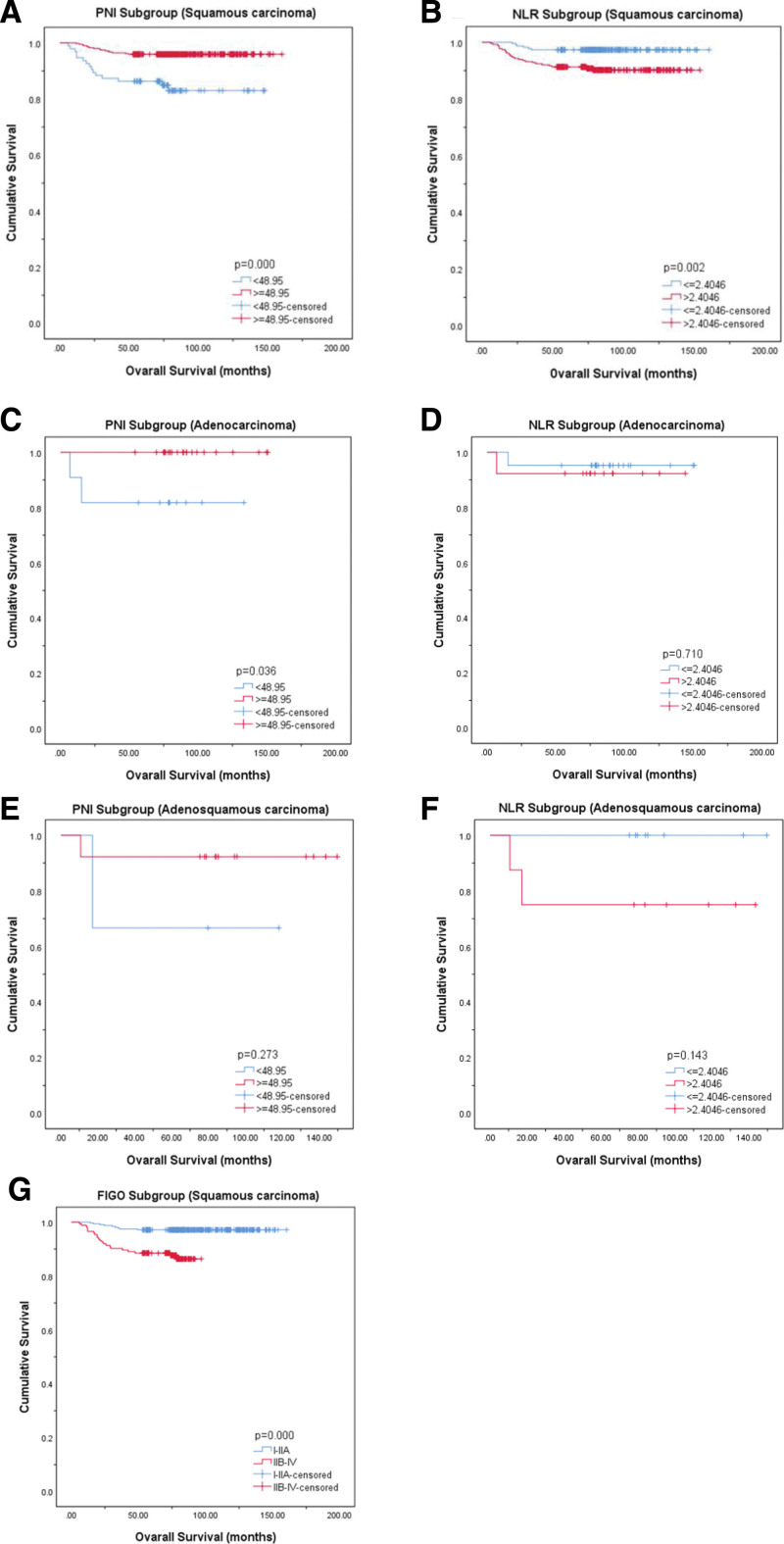
Analysis of KM curves. Overall survival in patients with: (A) cervical squamous carcinoma according to PNI; (B) cervical squamous carcinoma according to NLR; (C) cervical adenocarcinoma according to PNI; (D) cervical adenocarcinoma according to NLR; (E) cervical adenosquamous carcinoma according to PNI; (F) cervical adenosquamous carcinoma according to NLR; (G) cervical squamous carcinoma according to FIGO. FIGO = International Federation of Gynecology and Obstetrics, KM = Kaplan–Meier, NLR = neutrophil-to-lymphocyte ratio, PNI = prognostic nutritional index.

The results of the KM continuity study showed that patients with advanced PNI had significantly better OS than patients with low PNI (*P* < .001), while patients with low NLR had a considerable number of OS (*P* = .002) (Fig. 2A–B). The above results were limited to predicting the prognosis of cervical squamous cell carcinoma, but there was no significant difference between adenocarcinoma and adenosquamous cell carcinoma (Fig. 2C–F). At the same time, we found that, as shown in Figure 2G, the earlier the pathological stage, the longer the OS of the patient.

### 3.5. Establishment and validation of artificial neural network

**Figure 3.**
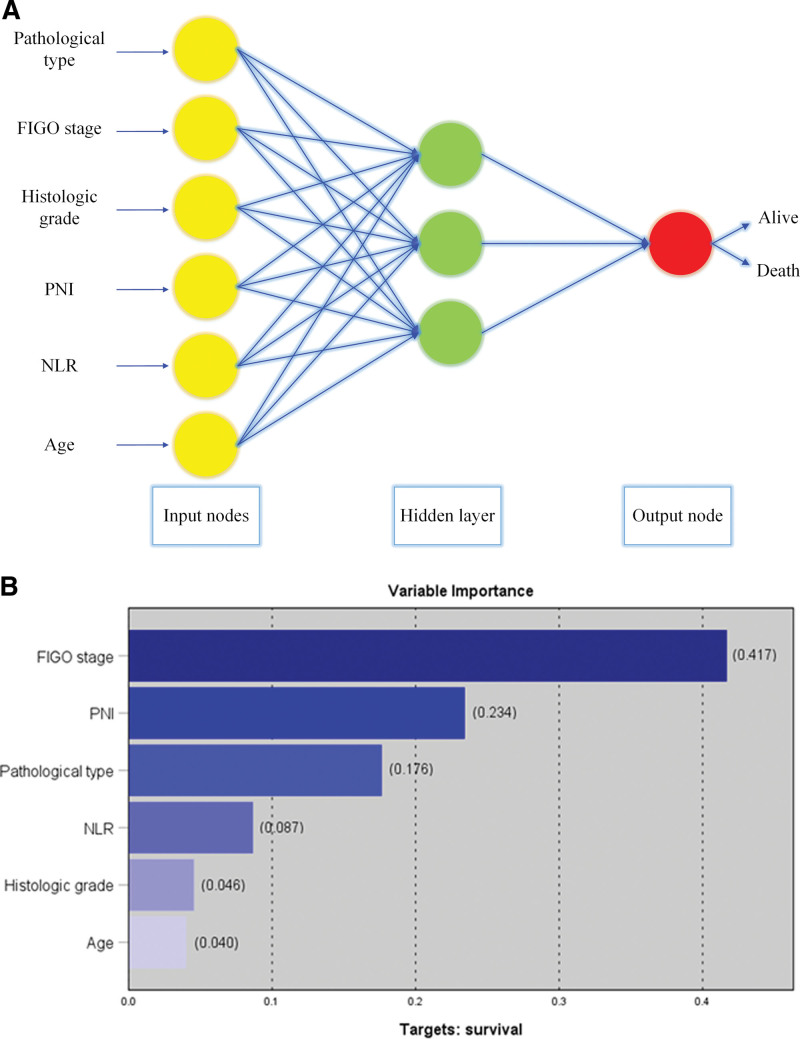
The construction of ANN. (A) Schematic representation of the ANN developed to predict survival for patients with cervical squamous carcinoma. (B) Importance of each variable in the ANN model. ANN = artificial neural network, FIGO = International Federation of Gynecology and Obstetrics, NLR = neutrophil-to-lymphocyte ratio, PNI = prognostic nutritional index.

The ANN model was established based on important risk factors identified by univariate and multivariate analysis (Fig. 3A). In the training queue, the prediction accuracy of ANN model is 95.4%, while in the validation queue, the prediction accuracy of ANN model is 93.95%. The scores of PNI and NLR in the ANN model are at a relatively favorable level (Fig. 3B).

## 4. Discussion

According to the analysis of global cancer epidemiological survey, cervical cancer is the main predisposing disease and cause of death in gynecological tumors. All over the world, the incidence rate and mortality rate of cervical cancer in adult women cannot be overlooked.^[1,2,21]^ According to the characteristics of cervical cancer, it can be divided into 3 common pathological types: squamous cell carcinoma, adenocarcinoma and adenosquamous cell carcinoma, accounting for 75% to 82%, 15% to 20%, and 3% to 5%, respectively.^[3,22]^ Due to its large number, younger incidence and complex pathological distribution, cervical squamous cell carcinoma has become the hotspot of clinical and scientific research.

We demonstrate that preoperative PNI and NLR are associated with the prognosis of patients with cervical squamous cell carcinoma and are independent risk factors for predicting cervical squamous cell carcinoma in this study. The establishment of ANN model shows that PNI and NLR can play a relatively important role in predicting the prognosis of patients with cervical squamous cell carcinoma, at least not worse than histologic grade. It can help clinicians timely and accurately identify high-risk patients and provide appropriate care choices.

Many studies have shown that the severity of systemic inflammation and poor nutritional status can predict survival.^[23,24]^ As early as the 19th century, some scholars first put forward the hypothesis that there are inflammatory cells in tumor tissue and there is an association between inflammation and tumor.^[25]^ There are not only a large number of inflammatory cells, but also high expression of inflammatory factors in tumor tissues. Inflammatory factors play an important role in the occurrence, development, invasion and metastasis of tumors.^[26]^ Previous studies have shown that neutrophils and lymphocytes are important components of innate and acquired immune system. Neutrophils are important mediators in the process of tumorigenesis and development. They can inhibit the activity of T lymphocytes and promote the occurrence and development of tumors by secreting a variety of cytokines (such as angiogenesis factor, IL-4, IL-1, etc).^[27]^ Patients with end-stage tumors are more likely to experience long-term severe nutritional consumption. These patients will then experience more serious weight and muscle loss. Long-term nutritional consumption is very easy to lead to malnutrition and decreased albumin levels. Albumin is recognized as an important substance for damage repair, and it can be used as a repair factor of a variety of antioxidants. The decrease of albumin will lead to the failure of antioxidants to repair, and then promote the progression of tumor.^[28,29]^

In recent years, PNI has been widely used to evaluate the nutritional status and prognosis of patients with different malignant tumors. AKGÜL et al conducted a meta-analysis of 637 patients with intrahepatic cholangiocarcinoma after radical resection. It was found that patients with PNI < 40 were more likely to suffer from multifocal diseases. Preoperative low PNI was associated with a more positive ICC phenotype. Patients with PNI ≥ 40 had better OS than patients with PNI < 40.^[30]^ HUA et al studied 380 patients with T1-2N1 breast cancer who underwent mastectomy. OS of patients with high PNI were significantly better than those with low PNI. Subgroup analysis of radiation therapy showed that OS in patients with high PNI was significantly better than those with low PNI, while OS in patients with low PNI was worse than those who did not receive radiotherapy.^[31]^ GENG et al retrospectively analyzed 321 cases of locally advanced or metastatic ductal adenocarcinoma of the pancreas. The results showed that low PNI was significantly associated with OS in patients with advanced pancreatic cancer. Multivariate analysis showed that PNI was an independent prognostic factor for OS.^[32]^ However, there is no study to prove the importance of PNI in the prognosis of cervical squamous cell carcinoma. Our results suggest that PNI is an independent prognostic factor of cervical squamous cell carcinoma. The improvement of PNI can significantly improve the OS rate of patients with cervical squamous cell carcinoma, but there is no significant difference between cervical adenocarcinoma and adenosquamous cell carcinoma.

NLR is a combined indicator of neutrophil and lymphocyte count, which reflects the balance between neutrophil related tumor inflammation and lymphocyte dependent anti-tumor immune response to a certain extent.^[33,34]^ Neutrophils stimulate capillary proliferation by secreting cytokines such as tumor necrosis factor and interleukin-1, which is conducive to tumor growth and metastasis.^[33,35]^ Lymphocytes play an anti-tumor immune role by inhibiting tumor cell proliferation and inducing apoptosis. The increase of NLR may represent the increase of tumor inflammatory response and the decrease of anti-tumor immune response.^[34]^ Huang et al have shown that the higher NLR level before treatment is associated with poor OS and short progression-free survival, which can be used as a predictor of poor prognosis in patients with ovarian cancer.^[36]^ A meta-analysis of malignant pleural mesothelioma by Chen et al showed that elevated NLR was associated with poor OS (HR = 1.48, 95% CI: = 1.16 ~ 1.89, *P* < .001). Elevated NLR may be a potential prognostic factor in patients with malignant pleural mesothelioma, and may be associated with histology as an effective clinical index for patient stratification.^[37]^ ZHOU et al conducted a meta-analysis of 37 articles from 43 pancreatic cancer cohort studies, and found that OS in patients with low NLR was longer. Subgroup analysis showed that low NLR was significantly associated with longer DFS; In addition, patients with low NLR had significantly smaller tumor size and higher degree of differentiation. Low NLR is a good predictor of OS and DFS in patients with pancreatic cancer.^[38]^ This study retrospectively analyzed the clinical data and serum data of 539 patients with cervical cancer. It was found that NLR can be used as an independent predictor of the prognosis of patients with cervical squamous cell carcinoma.

In this study, PNI and NLR were combined to evaluate and predict the subordinate relationship with the prognosis of cervical squamous cell carcinoma. Cox regression univariate analysis showed that the age of patients, pathological type, FIGO stage, histological grade, PNI and NLR were statistically significant. At the same time, in order to avoid the influence of individual variables, we included the above 6 indexes into a multivariable study. It was found that pathological type, FIGO stage, preoperative PNI and NLR were independent prognostic factors for patients with cervical squamous cell carcinoma. The lower the FIGO stage, the higher the PNI value and the lower the NLR value, the longer the survival time of patients with cervical squamous cell carcinoma.

This study has some limitations. Although we strictly limited the inclusion and exclusion criteria, serum albumin, neutrophil and lymphocyte counts may still be affected by potential confounding factors such as smoking and drinking. In addition, this study is a small sample, single center and retrospective study, which may have selection bias. The significance and value of the selection of PNI and NLR bounds and the evaluation of prognosis need to be verified by a large sample prospective study.

In conclusion, our results show that PNI and NLR scores are related to the prognosis of patients with cervical squamous cell carcinoma. PNI and NLR can be obtained by blood routine examination, which is efficient, economical, noninvasive and highly operable. PNI and NLR scores may help clinicians to formulate effective individualized treatment plans and improve the survival rate and prognosis of patients with cervical squamous cell carcinoma.

## Author contributions

**Conceptualization:** Aiqin He, Xiaohao Li.

**Data curation:** Aiqin He.

**Formal analysis:** Xiaohao Li.

**Funding acquisition:** Aiqin He.

**Investigation:** Chaoyang Ou.

**Methodology:** Chaoyang Ou.

**Project administration:** Aiqin He.

**Resources:** Aiqin He.

**Software:** Xiaohao Li.

**Validation:** Aiqin He.

**Visualization:** Xiaohao Li.

**Writing – review & editing:** Aiqin He.

**Writing – original draft:** Xiaohao Li.
